# ConvNeXt-MHC: improving MHC–peptide affinity prediction by structure-derived degenerate coding and the ConvNeXt model

**DOI:** 10.1093/bib/bbae133

**Published:** 2024-04-01

**Authors:** Le Zhang, Wenkai Song, Tinghao Zhu, Yang Liu, Wei Chen, Yang Cao

**Affiliations:** College of Computer Science, Sichuan University, Chengdu 610065, China; College of Computer Science, Sichuan University, Chengdu 610065, China; College of Computer Science, Sichuan University, Chengdu 610065, China; Nuclear Power Institute of China, Chengdu 610213, China; Center of Growth, Metabolism and Aging, Key Laboratory of Bio-Resource and Eco-Environment of Ministry of Education, College of Life Sciences, Sichuan University, No. 29 Wangjiang Road, Chengdu 610065, China; Innovative Institute of Chinese Medicine and Pharmacy, Chengdu University of Traditional Chinese Medicine, Chengdu 611137, China; Center of Growth, Metabolism and Aging, Key Laboratory of Bio-Resource and Eco-Environment of Ministry of Education, College of Life Sciences, Sichuan University, No. 29 Wangjiang Road, Chengdu 610065, China

**Keywords:** MHC, peptide–MHC binding prediction, neoantigen, T cell epitope, ConvNeXt

## Abstract

Peptide binding to major histocompatibility complex (MHC) proteins plays a critical role in T-cell recognition and the specificity of the immune response. Experimental validation such peptides is extremely resource-intensive. As a result, accurate computational prediction of binding peptides is highly important, particularly in the context of cancer immunotherapy applications, such as the identification of neoantigens. In recent years, there is a significant need to continually improve the existing prediction methods to meet the demands of this field. We developed ConvNeXt-MHC, a method for predicting MHC-I-peptide binding affinity. It introduces a degenerate encoding approach to enhance well-established panspecific methods and integrates transfer learning and semi-supervised learning methods into the cutting-edge deep learning framework ConvNeXt. Comprehensive benchmark results demonstrate that ConvNeXt-MHC outperforms state-of-the-art methods in terms of accuracy. We expect that ConvNeXt-MHC will help us foster new discoveries in the field of immunoinformatics in the distant future. We constructed a user-friendly website at http://www.combio-lezhang.online/predict/, where users can access our data and application.

## INTRODUCTION

The binding of short peptide fragments to major histocompatibility complex (MHC) molecules is a crucial and discriminating step in initiating an adaptive immune response [1]. Accurate identification of peptides that can bind to specific MHC molecules is of paramount importance for understanding the underlying mechanisms and discovering immunogenic epitopes, such as neoantigens [2]. Moreover, promiscuous MHC epitopes that can bind to multiple MHC alleles are critical candidates for the development of vaccines. There are two distinct classes of MHC molecules: class I (MHC-I) and class II (MHC-II) [3]. To detect endogenous peptides, MHC-I is present in all cells except red blood cells, while MHC-II is found mainly on B cells, dendritic cells, macrophages and certain antigen-presenting cells, where it binds only to exogenous peptides [4].

Experimental validation of the peptides binding to specific MHC molecules is not only extremely broad but also involves both time- and cost-consuming biological experiments [5]. Therefore, precise identification of such peptides using computational methods is a central challenge in immunoinformatics [6]. In traditional allele-specific methods, binding data for only one allele can be used to train a model, and the model can be applied only to predict peptide binding to the allele. Methods, e.g. NetMHC [7], SMM for MHC-I [8], SMM-align [9], NN-align [10] and RTA for MHC-II [11], are all outstanding allele-specific methods with decent accuracy. To address the problem of predicting peptides binding to MHC molecules with only a small number of experimentally obtained binders, a series of computational approaches, called panspecific methods, have been developed and have received intense interest [12]. Pan-specific methods use pseudo sequences instead of MHC alleles as inputs and attempt to predict binders. The pan-specificity predictors define the so-called pseudo sequence, which is the residue of the MHC molecule within a distance of 4.0 Å between any pair of atoms from the MHC and the bound peptide taken from the known peptide–MHC crystal structures [6]. The pan-specificity predictor uses pseudo sequences as a feature of the machine learning process to classify a peptide as a binder or non-binder [13]. Constructing the pan-specificity predictor generally involves the following four major steps: (i) collecting training datasets that experimentally verify the binding ability between the MHC and peptide [14], (ii) extracting pseudo sequences encoding from the peptide and MHC sequences [6], (iii) choosing the best-performing machine learning algorithm and training the corresponding machine learning model [7] and (iv) optimizing and evaluating the model and its performance.

To date, multiple pseudo sequences have been reported. NetMHCPan [15] is a well-established pan-specific binding affinity prediction algorithm that reduces the MHC sequence to a pseudo sequence 34 in length that includes the following residues: 7, 9, 24, 45, 59, 62, 63, 66, 67, 69, 70, 73, 74, 76, 77, 80, 81, 84, 95, 97, 99, 114, 116, 118, 143, 147, 150, 152, 156, 158, 159, 163, 167 and 171. MHCflurry2.0.1 [16] uses the same 34 peptide-contacting residues as well as 3 additional residues (91, 102 and 199). Moreover, MixMHCpred [17] employs 32 peptide-contacting residues as the pseudo sequence. These pseudo sequences have shown advantages over other allele-specific methods. However, the pseudo sequences and peptides are linked together, without distinguishing the residues of MHC from those of peptides. What is more, the information of pair-wise interactions between the MHC and peptide are ignored [18]. These residue interactions are essential for physical binding, suggesting that it could be beneficial to explicitly consider them in the prediction model [19, 20].

The development of computational methods that accurately and efficiently provide prediction for peptide–MHC binding affinity can greatly contribute to immunotherapies and vaccine development. Despite the existence of various methods, none of them fully satisfies the requirements for biomedical practice. Particularly, although state-of-the-art methods extract various information from MHC and peptide sequences and structures, most of these methods rely on simple feature fusion models to capture MHC–peptide relationships. To address this challenge, we aimed to develop a comprehensive MHC-I pseudo sequence by incorporating features of pair-wise residue interactions between MHC-I and peptides as well as more advanced deep learning techniques to increase predictive accuracy. Three innovations are proposed for this study.

First, a new pseudo-sequence coding method, degenerate encoding, was developed by encoding the pair-wise residues interacted between pseudo sequences and peptides together in two-dimensional arrays. Hence, the pair-wise interactions are explicitly embedded in the model.

Second, in recent studies, deep learning has exhibited powerful capabilities for extracting latent patterns within biological sequences [21–33]. Our work takes advantage of the recently developed deep learning model ConvNeXt [34] for prediction. ConvNeXt is an improved convolutional neural network (CNN) model that can handle a wide range of vision tasks and has achieved state-of-the-art performance in many benchmark datasets. Our work also incorporated transfer learning [35–37] and semi-supervised learning to enhance its power.

Third, an online prediction platform was developed for predicting and visualizing the binding of MHC-I molecules to peptides via related data sharing, which can help users make more intuitive and convenient predictions, improving antigen screening efficiency [38].

In conclusion, we developed a new method, namely, ConvNeXt-MHC, to enhance MHC-I-peptide affinity prediction. ConvNeXt-MHC can predict the presentation of antigens (ConvNeXt-MHC_AP) and binding affinity (ConvNeXt-MHC_BA). According to our comprehensive benchmark tests, ConvNeXt-MHC outperforms state-of-the-art methods in terms of accuracy, F1-score, MCC and binding affinity correlation. Notably, ConvNeXt-MHC exhibits remarkably enhanced ability to predict 8-mer peptides. Further analysis illustrates the degenerate encoding approach is the major contributor of the improvement compared to the traditional approaches. To facilitate the use of ConvNeXt-MHC, we constructed a user-friendly online server which is freely available for researchers world-wide. The details of the method are described in the following sections.

## MATERIALS AND METHODS

### Datasets

The dataset comprises two types of data for a given MHC allele: mass spectrometry (MS)–identified peptide [39] and peptide–MHC affinity (AF) data. AF data measure the ‘half maximum inhibitory concentration’ (IC50), where a lower value indicates stronger affinity [40]. The data can be transformed into a continuous target scale (log50k) in the range of [0,1] according to Supplementary Table 4. We obtained MS data from recently published reports and AF data from the Immune Epitope Database (IEDB) [41]. Overall, the collected data were divided into five sets as described below.

(i) MS training set for ConvNeXt-MHC_AP: 1 048 575 records with 206 515 positive data points and 842 060 negative data points. These sequences were obtained from the SysteMHC Atlas project [42] and from TransPHLA-AOMP [43], as well as from studies by Sarkizova *et al*. [44], Abelin *et al*. [45] and Karosiene *et al*. [46].
(ii) MS test set for ConvNeXt-MHC_AP: 6342 records with 3133 positive data points and 3209 negative data points were obtained from the works of Pearson *et al*. [47] and Bulik-Sullivan *et al*. [46].
(iii) AF training set for ConvNeXt-MHC_BA: 210 509 records obtained from the IEDB database [41]. To obtain low-affinity peptides, we used NullSeq [48] to generate 38 633 peptides randomly, which were labeled with the predicted affinities estimated by MHCflurry2.0 and NetMHCpan4.0 (predicted IC50 > 1000 nM).
(iv) AF test set for ConvNeXt-MHC_BA: 887 binding affinity data points obtained from the testing dataset of NetMHCpan4.1 [15].
(v) Additional AF test set for ConvNeXt-MHC_BA: 212 records obtained from weekly data between Oct. 2021 and June 2023 in the IEDB database [41]

In order to ensure the integrity of our analysis, we took steps to address any potential overlaps between the MS testing dataset and the MS training dataset. We identified and removed any shared data points from the testing dataset, and we also excluded non-*Homo sapiens* data from both sets. This process was carried out with the same level of diligence for the AF testing and training datasets, ensuring a consistent approach across all datasets used in our study.

Pseudo sequence: We collected a total of 552 MHC-I-peptide structure files from the PDB database [49] and eliminated those with undefined amino acids and non-9-mer peptides. 354 pMHC-I structures were ultimately used to determine the pseudo sequence of MHC-I. The details are described in Supplementary Method 2 , Supplementary Method 6, Supplementary Figure 1 and Supplementary Figure 2.

### Degenerate coding

The traditional pseudo sequences are designed to capture the sequence features of MHC alleles. Each residue of the sequence was subsequently transformed into a 21-dimensional vector using the BLOSUM62 substitution matrix or one-hot codes. For example, MHCflurry uses BLOSUM62, and NetMHCpan 4.0 uses BLOSUM50 to encode pseudo sequences [50, 51]. Such models lack information on residue contacts between peptides and MHCs, but it is key for peptide–MHC binding.

To incorporate contact information between MHC-I molecules and 9-mer peptides in their three-dimensional structure, we designed two-dimensional image-like arrays (ILAs) to represent MHC-I pseudo sequences and 9-mer peptide sequences. This ILA did not explicitly include the pseudo sequence but interlocked at the position of the peptide (Figure 2). Thus, we call this degenerate coding. The ILA has a width of 9, representing the length of the 9-mer peptide; a height of 20, representing the 20 amino acid types in the pseudo sequence; and a depth of 21, representing the contact marks and 20 amino acids of the peptide. As shown in Figure 2A, we used Supplementary Method 3.1 to generate a pseudo sequence (20*9*1) for each MHC, where 1 or 0 represents that the 9-mer peptide is in contact with this type of residual amino acid or not (Supplementary Method 3.1 and Supplementary Figure 3).

### Development of the deep learning model

ConvNeXt-MHC was designed by the following classic ConvNeXt model [34], which is illustrated in Supplementary Figure 4. The specific operation of the model is shown in Equations (1) and (2) [52].


(1) \begin{equation*} DepthConv{\left({V}_c,{X}_{\left[:,:,c\right]}\right)}_{\left(i,j\right)}=\sum_{k,l}^{K,L}{V_c}_{\left(k,l\right)}\odot{X_{\left[:,:,c\right]}}_{\left(i+k,j+l\right)} \end{equation*}

(2) \begin{align*} & WiseConv{(X)}_{\left(i,j\right)} \nonumber \\ &=\left[ DepthConv{\left({V}_1,{X}_{\left[:,:,1\right]}\right)}_{\left(i,j\right)};\dots; DepthConv{\left({V}_C,{X}_{\left[:,:,C\right]}\right)}_{\left(i,j\right)}\right] \end{align*}

We use $\odot$ to denote the element-wise product, where $i,j,k$ and $l$ are the corresponding indices. Among them, $X=\left[{X}_{\left[:,:,1\right]};\dots; {X}_{\left[:,:,C\right]}\right]$, where matrix $X$ represents the input matrix. $C$ represents the number of channels in the third dimension of the input matrix $X$ and $c\in C$. ${V}_{\mathrm{c}}$ represents the convolution kernel corresponding to the $c$ channel, while $K$ and $L$ represent the convolution kernel sizes, $k\in K$, $l\in L$ and $i$ and $j$ represent the positions of the first two dimensions of $X$; $DepthConv$ represents depth convolution calculation; $WiseConv$ represents point-by-point convolution calculation. Please see the nomenclature in Supplementary Table 1.

#### Weight initialization for the attention mechanism

As shown in Figure 3A, we employed an attention mechanism to determine the impact of the amino acid type of the residue on the 9-mer peptide by adding neurons to each layer of the ILA. Unlike in previous studies [24, 53], we initialize the weights of the attention layer via transfer learning and prior knowledge [Equation (3)] and use the attention block to obtain the attention for each layer matrix [Equation (4)]. Adding attention to the matrix results in a hybrid attention matrix X’ [Equation (5)], which can increase the accuracy and generalizability of the predictive model (Supplementary Method 4).


(3) \begin{equation*} init\_{weight}_h=\frac{frequency_{Amio\_ acid}\left[h\right] in\left({frequency}_{Amio\_ acid}\left[h\right]\right)}{\mathit{\operatorname{Max}}\left({frequency}_{Amio\_ acid}\left[h\right]\right)\!-\!\mathit{\operatorname{Min}}\left({frequency}_{Amio\_ acid}\left[h\right]\right)} \end{equation*}

(4) \begin{equation*} {a}_h= Conv1{D}_h\left({X}_{\left[h,:,:\right]}, kernal\_ size=W, filter=1\right) \end{equation*}

(5) \begin{equation*} {X}^{\prime }=\left[{a}_1I{X}_{\left[1,:,:\right]},\dots, {a}_HI{X}_{\left[H,:,:\right]}\right] \end{equation*}

${frequency}_{Amio\_ acid}\left[h\right]$ represents the frequency of 20 amino acids, with $h$ being 1 of the 20 amino acids. The initialization parameter corresponding to the number of layers of amino acid $h$ is $init\_{weight}_h$. Equation (3) helps us obtain the network initialization value $init\_{weight}_h$ and initializes the corresponding neurons $Conv1{D}_h$. Among them, $X=\left[{X}_{\left[1,:,:\right]},\dots, {X}_{\left[h,:,:\right]}\right]$, $I$ is the identity matrix, ${a}_h$ represents the attention value and ${X}^{\prime }$ is an input matrix mixed with attention for subsequent network processing.

#### Training data augmentation via semi-supervised learning

Utilizing the semi-supervised method to generate AF data with similar characteristics to MS data can improve prediction accuracy [54]. As shown in Figure 3B, we adopt data augmentation based on semi-supervised learning to convert the MS data into AF data to pretrain our deep learning model (Supplementary Method 5).

The transformation rule between the IC50 of AF data and the discrete values of antigen presentation is shown in Equation (6) [55].


(6) \begin{equation*} label=\left\{\begin{array}{@{}ll} 1, & IC50<500\mathrm{nM}\\{}0, & IC50\ge 500\mathrm{nM}\end{array}\right. \end{equation*}

#### Implementation of the overall scheme

The overall scheme was implemented by following steps. Firstly, we extracted the pseudo sequence corresponding to the input MHC-I allele as described in Supplementary Method 3.1. Secondly, we generated the ILAs for both the 9-mer peptide and the MHC-I pseudo sequence, following the procedures outlined in Supplementary Methods 3.2 and 3.3. Thirdly, attention blocks and ConvNeXt networks were constructed, as illustrated in Supplementary Figure 4. The initialization of the attention matrix parameters is detailed in Supplementary Method 4. Next, we employed semi-supervised learning techniques, as explained in Supplementary Method 5. Lastly, we established the prediction models for ConvNeXt-MHC_AP and ConvNeXt-MHC_BA based on the aforementioned steps.

## RESULTS

**Figure 1:**
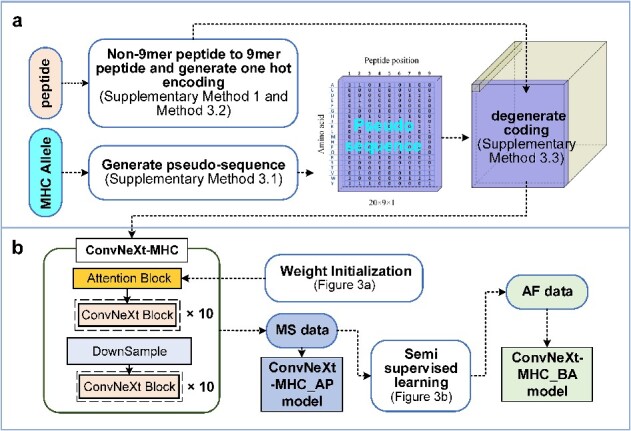
The architecture of the ConvNeXt-MHC. (**A**) The process of degenerate coding. (B) ConvNeXt-MHC model.

ConvNeXt-MHC employs degenerate coding to characterize the binding modes between peptides and MHC-I molecules and utilizes the semi-supervised learning method of the ConvNeXt model to increase its predictive power (Figure 1). The tool can predict the presentation of antigens (ConvNeXt-MHC_AP) and the binding affinity (ConvNeXt-MHC_BA). Our comprehensive benchmark tests are described in the following sections.

**Figure 2:**
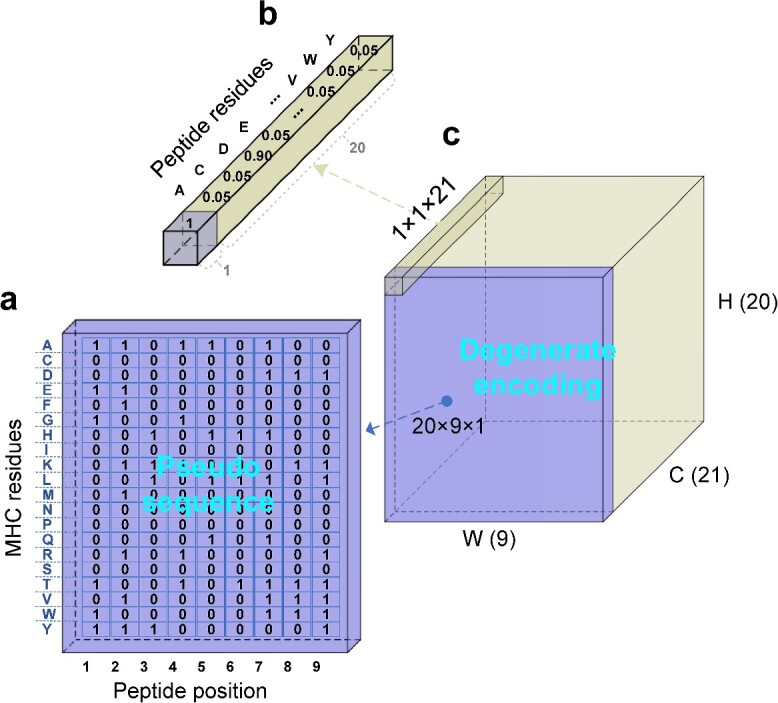
The data structure of degenerate coding. The ILA has 21 channels with the first channel representing the contact information between the 9-mer peptide and the amino acid types of the MHC. The remaining 20 channels used a one-hot encoding method to represent the specific amino acid type of the 9-mer peptide. (**A**) Pesudo sequence: it is generated by Supplementary Method 3.1. Among them, 1 represents the presence of the corresponding amino acid residue in the polypeptide position, and 0 represents its absence. (**B**) Peptide residues: it shows that the contact amino acid type of the 9-mer peptide was 0.90; otherwise, 0.05. (**C**) The degenerate coding of peptide and pseudo sequence.

**Figure 3:**
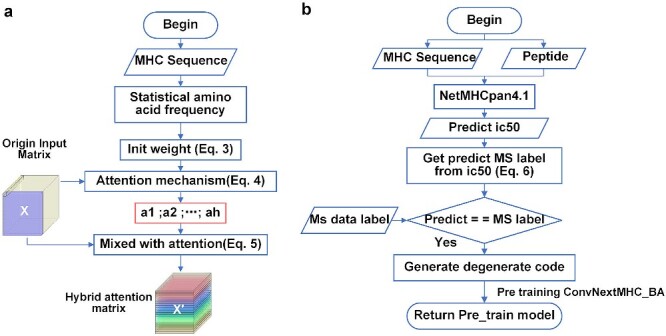
The workflow of the ConvNeXt-MHC model. (**A**) Attention mechanism and its module for initialization. (**B**) Data augmentation via semi-supervised learning.

### The performance of ConvNeXt-MHC_AP on the MS test set

**Figure 4:**
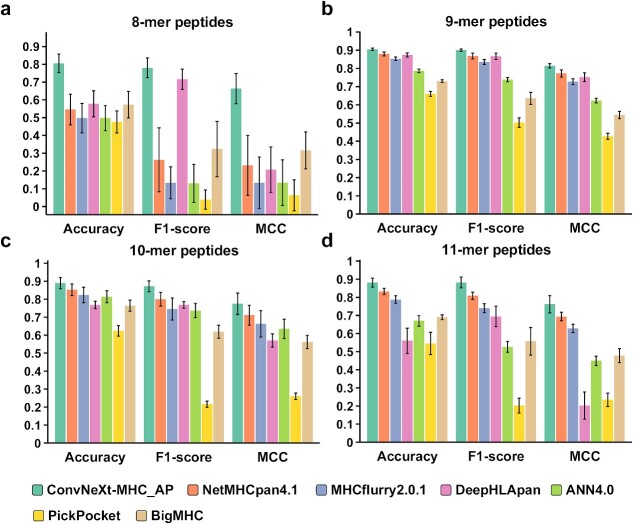
Benchmarking results for ConvNeXt-MHC_AP and other state-of-the-art methods. (**A**) Results for 8-mer peptides. (**B**) Results for 9-mer peptides. (**C**) Results for 10-mer peptides. (**D**) Results for 11-mer peptides.

To evaluate the performance of ConvNeXt-MHC in the case of the antigen presentation predictive mode ConvNeXt-MHC_AP, we conducted benchmark tests using the MS test set (2.1 datasets). We compared our method with state-of-the-art methods, including PickPocket1.1 [56], ANN4.0 [7], NetMHCpan4.1 [57], MHCflurry2.0 [16], DeepHLApan [25] and BigMHC [58] using the same benchmark data. The evaluation metrics included accuracy, F1-score and the Matthews correlation coefficient (MCC), which are well-established criteria for assessing prediction model performance, especially for yes or no questions (detailed descriptions are provided in Supplementary Table 4). The testing data were categorized into four groups based on peptide length. Overall, ConvNeXt-MHC_AP outperformed the other methods, achieving the highest accuracies, F1-scores and MCCs across all four categories (Figure 4). While the differences between these methods were marginal for 9-mer peptides, ConvNeXt-MHC_AP demonstrated significant advantages over the other methods, particularly showing more than 30% improvement in 8-mer peptides (Figure 4A). The success of these 8-mer peptides could be attributed to the fact that 8-mer peptides share highly similar binding modes with 9-mer peptides, although the number of available training data for 8-mer peptides is much smaller than that for 9-mer peptides (for which the sample numbers are 7397 versus 142 601). Thus, the encoded binding information compensates for the deficiency of 8-mer peptides in the training data. These results highlight the effectiveness of our degenerate encoding and ConvNeXt model in predicting non-9-mer peptides, which has been a challenging task in this field. For a more detailed explanation and comprehensive statistical tests, please see Supplementary Table 5 and the process is outlined in Supplementary Figure 7.

### The performance of ConvNeXt-MHC_BA on AF test sets

**Figure 5:**
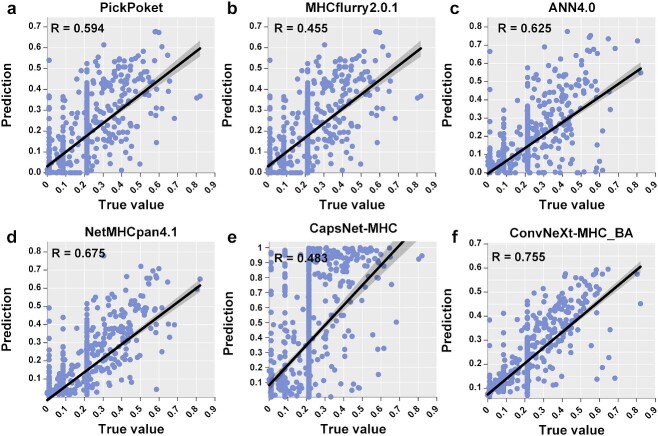
The performance of, PickPocket (**A**), MHCflurry2.0.1 (**B**), ANN4.0 (**C**), NetMHCpan4.1 (**D**), CapsNet-MHC (**E**) and ConvNeXt-MHC_BA (**F**) on the peptide–MHC interaction test set. The Pearson correlation coefficients are shown in the top left corner.

Predicting the binding affinity of peptides is more challenging than predicting antigen presentation mode using the MS dataset, as it requires quantifying the strength of the binding. To assess the performance of ConvNeXt-MHC in this context (ConvNeXt-MHC_BA), we conducted benchmark tests using the AF test set (2.1 datasets). We also compared our method with state-of-the-art methods that support binding affinity prediction, namely, PickPocket, ANN4.0, NetMHCpan4.1, MHCflurry2.0.1 and CapsNet-MHC [19]. The results are presented in scatter plots (Figure 5), depicting the correlation between the experimentally determined true values and the predicted values of the five methods. ConvNeXt-MHC_BA exhibited a strong correlation with the true and predicted values. The Pearson correlation coefficients for PickPocket, MHCflurry2.0.1, ANN4.0, NetMHCpan4.1, CapsNet-MHC and ConvNeXt-MHC_BA were 0.594, 0.455, 0.625, 0.675, 0.483 and 0.755, respectively. Furthermore, the squared error of ConvNeXt-MHC_BA was significantly less than that of the other methods (Supplementary Table 6). To further evaluate the performance, we employed an additional AF test set, which was collected from the recently released weekly IEDB data. The results showed that the squared error of ConveNeXt-MHC_BA was significantly less than that of the other comparisons (Supplementary Figure 8).

Once again, ConvNeXt-MHC_BA demonstrated remarkable advantages over the other methods in the benchmark tests.

### Degenerate coding outperforms well-established coding methods

This success can be largely attributed to the effectiveness of degenerate coding and the ConvNeXt model. To reveal the contribution of degenerate coding, we implemented a simple CNN prediction model that can perform neck-to-neck comparisons between degenerate coding and other well-established coding approaches, including the pseudo sequence coding described in NetMHCpan4.1, MHCflurry2.0.1 (The same coding as CapsNet-MHC), DeepHLApan and ANN. These models are referred to as DE-CNN, NetMHCpan-CNN, MHCflurry-CNN, DeepHLApan-CNN and ANN-CNN, respectively. The specific positions of the three pseudo sequences are outlined in Supplementary Table 2.

To ensure a fair and meaningful comparison, we trained and tested all the models, namely, DE-CNN, NetMHCpan-CNN, MHCflurry-CNN, DeepHLApan-CNN and ANN-CNN, by identical CNN structures and a rigorous 5-fold cross-validation technique [39, 59]. Supplementary Table 3 and Supplementary Figure 6 provides comprehensive information regarding the training and testing process.

Through extensive experiments conducted using 5-fold cross-validation on the MS training set, we obtained average accuracies of 0.953, 0.902, 0.928, 0.923 and 0.882 for DE-CNN, NetMHCpan-CNN, MHCflurry-CNN, DeepHLApan-CNN and ANN-CNN, respectively (Table 1). These results illustrate the statistically significant improvements of DE-CNN over the other traditional approaches. Furthermore, Supplementary Figure 5 presents detailed data on accuracy, F1-score and MCC, providing further evidence of the advantages conferred by degenerate encoding.

**Table 1:** The Shapiro test and *t*-test for encoding methods of DE-CNN, NetMHCPan, MHCflurry, ANN and DeepHLApan after 5-fold cross-validation

Encoding method	Average accuracy	Std	Shapiro	T-test (*P*-value)
MHCflurry-CNN	0.928	0.004	0.050	0.001
NetMHCpan-CNN	0.902	0.004	0.586	0.000
DE-CNN	0.953	0.008	0.710	1.000
DeepHLApan-CNN	0.923	0.002	0.457	0.001
ANN-CNN	0.882	0.008	0.431	0.000

In order to gain a deeper understanding of the enhancements brought about by ConvNeXt compared to CNN, we conducted a performance comparison between ConvNeXt-MHC and DE-CNN. These models only differ in terms of the machine learning algorithm utilized. Supplementary Tables 7 and 8 present the results, showing that ConvNeXt-MHC outperforms DE-CNN by 1.1% in accuracy, 1.04% in F1-score and 1.04% in MCC. These findings demonstrate the contribution of ConvNeXt to our methodology.

Collectively, these experimental findings solidify the superiority of our degenerate coding method over traditional approaches.

### ConvNeXt-MHC online platform

**Figure 6:**
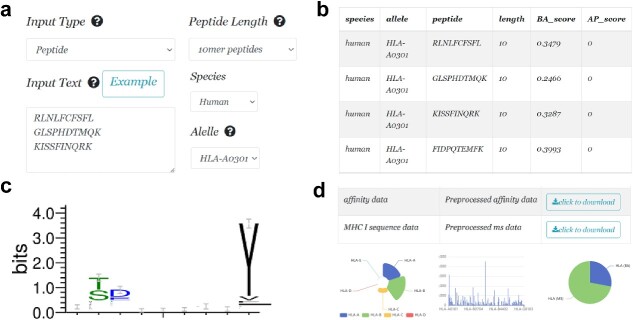
ConvNeXt-MHC online platform. (**A**) The data entry, (**B**) The results of BA and AP prediction, (**C**) The sequence logo of peptides and (**D**) The statistics of the data used in this model.

To facilitate the use of ConvNeXt-MHC, we developed a user-friendly online predictive platform at http://www.combio-lezhang.online/predict/. This platform provides a range of functionalities (Figure 6). This allows users to conveniently submit their data and promptly present a comprehensive list of predictive BA and AP values using the power of ConvNeXt-MHC. For a more detailed analysis, an insightful motif analysis was performed [60] for different peptide sequences that exhibit allele-binding properties. This analysis provides users with a deeper understanding of the underlying patterns and characteristics of these peptides, paving the way for further investigation and discovery. Furthermore, it caters to users’ need for statistical analysis and empowers them with the ability to download the MHC-I data that were collected for our study. By seamlessly integrating these functionalities, ConvNeXt-MHC online predictive platform serves as a convenient tool for researchers and practitioners alike, enabling them to make informed decisions and expedite their research in the field of MHC-I binding prediction.

## DISCUSSION

The MHC-I plays a vital role in presenting signals to the immune system for detecting and eliminating infected or cancerous cells. The development of computational methods that accurately and efficiently provide comprehensive explanations for predicting peptide–MHC binding is of paramount importance in advancing immunotherapies and vaccine development. However, despite the existence of several methods, none of them fully meet the demanding requirements. In this work, we propose a novel semi-supervised model for peptide presentation by MHC class I molecules to improve MHC-I peptide affinity prediction. Our model incorporates degenerate coding, explicitly capturing the interaction between MHC and peptides. Furthermore, we leverage the ConvNeXt model to enhance our method. Extensive experiments on MHC-I-peptide binding prediction demonstrated that ConvNeXt-MHC outperforms state-of-the-art methods in terms of accuracy, F1-scores and MCCs on our benchmark datasets. Notably, ConvNeXt-MHC exhibited a markedly enhanced ability to predict non-9-mer peptides.

Although ConvNeXt-MHC showed advanced performances, there are still some shortcomings that we aim to address in our subsequent work. Firstly, our protocol relies on a machine-learning model, which inherently depends on the size and quality of the training data. In our tests, we did observe a noticeable decrease in prediction accuracy for a small number of MHC alleles that had infrequent occurrences in the training data. Secondly, the degenerate coding approach relies on a static residue list for pair-wise interactions between MHC-I and peptides, without considering any alterations that may occur due to variations in residue types. As a result, the static residue list may introduce errors in certain cases when the sizes and physicochemical properties vary, as the real pair-wise interactions can change. Thirdly, our method’s running speed is comparatively slower compared to popular methods like NetMHCpan4.1 and MHCflurry2.0. This is primarily due to the architecture of our model, which affects the overall computational efficiency.

To facilitate the utilization of ConvNeXt-MHC, we developed a user-friendly web server. This web server offers a comprehensive One-Stop service, which includes predicting binding affinities, peptide residue preferences, MHC allele distributions and other valuable insights. By leveraging ConvNeXt-MHC through this web server, researchers will be empowered to analyze large-scale epitopes, fostering new discoveries in the dynamic field of immunoinformatics.

**Key Points**

- To encode the pair-wise interactions between the residues of peptides and MHCs, a new pseudo sequence coding method was developed.
- ConvNeXt-MHC integrates transfer learning and semi-supervised learning methods into a cutting-edge deep learning framework ConvNeXt.
- Comprehensive benchmark results demonstrate that ConvNeXt-MHC outperforms state-of-the-art methods in terms of accuracy.
- ConvNeXt-MHC is implemented in a user-friendly web server that is freely available at http://www.combio-lezhang.online/predict/.

## Supplementary Material

Supplementary_v3_bbae133

## Data Availability

The source codes are available at http://www.labshare.cn/ConvNeXt-MHC/. See Supplementary Materials and Methods for more details of materials and methods.

## Author Biographies

**Le Zhang** is currently a professor in the College of Computer Science, Sichuan University, China. More information can be found at the website of his group: http://120.78.69.27/zhangle/index_English.html#.

**Wenkai Song** is currently a master student at College of Computer Science, Sichuan University, China. His research interest focuses developing methods for AI-based methods for computational biology.

**Tinghao Zhu** graduated from the College of Computer Science, Sichuan University, China. She is currently a researcher at the Nuclear Power Institute of China.

**Yang Liu** is currently a PhD candidate at College of Life Sciences, Sichuan University, China. He mainly focuses on developing methods for sequence analysis and computer-aided drug design.

**Wei Chen** is currently a professor in Chengdu University of Traditional Chinese Medicine, China. More information can be found at the website of his group: https://chenweilab.cn/.

**Yang Cao** received his PhD from Institute of Biophysics, Chinese Academy of Sciences. He is currently an associate professor at College of Life Sciences, Sichuan University. His major research interests include developing new methods for computer-aided drug and protein design.
