# Supplementary material for: ConvNeXt-MHC: improving MHC–peptide affinity prediction by structure-derived degenerate coding and the ConvNeXt model
Source: Brief Bioinform. 2024 Apr 1;25(3):bbae133. doi: 10.1093/bib/bbae133 (PMC10985285; doi:10.1093/bib/bbae133)
Supplement: Supplementary_v3_bbae133 [file supplementary_v3_bbae133.pdf]

ConvNeXt-MHC: improving MHC-peptide affini-ty prediciton by structure-derived degenerate coding and the ConvNeXt model

Supplementary

Supplementary Method 1：Non-9-mer to 9mer conversion algorithm

Firstly, Non-9mer to 9mer conversion algorithm listed all possible 9mer peptides for each non-9mer peptide. And then, we employed NetMHCpan4.1[1] to have the affinity predictive score for each possible 9mer peptide. Finally, we selected such 9mer peptide to represent the corresponding non-9mer peptide that has the highest affinity predictive score.

Method 1 Non-9-mer to 9mer conversion algorithm

Input: Peptide sequences, e.g. 8, 10, 11, 12, 13, 14, 15 in length

Output: Representative 9mer-peptide sequence

```
n ← The length of peptide
V = [] ← Collection of all possible 9mer peptide sequences
if n == 8 do
    for i = 1 to 9 do
        v = peptide    ← A possible 9mer peptide initialized to peptide sequence
        add(v, i, "-")   ← Add a vacancy of "-" at the i th postion
        append(V, v)   ← Appended v to the collection of V
    end for
end if
if n!=8 do
    for i = 1 to n do
        for k = 1 to 9 do
            v = []    ← A possible 9mer peptide
            append(v, peptide[(i+k)%n])  ← Add amino acid of peptied   to v
        end for
        append(V, v)   ← Appended v to the collection of V
    end for
end if
highest_score = 0 , highest_9mer = ""   ← Initialize AF prediction highest value and best 9mer peptide
NetMHCpan_ Vec = [] ← NetMHCpan4.1 input matrix array
for v in V do
```

M = NetMHCpan_Input_Matrix(*v*) ← Generate NetMHCpan4.1 matrix with respect to *v*
AF_score = NetMHCpan4.1(*M*) ← Get the AF predicted score from NetMHCpan4.1
if *AF_score* > *highest_score* **do**
highest_9mer = *v* ← Record the 9mer peptides corresponding to the highest predicted AF value
return *highest_9mer*

Supplementary Method 2: MHC I pseudo sequence site distribution algorithm

We obtained MHC I pseudo sequence site distribution information by computing the distance between MHC I molecules and peptides, aligning MHC I sites in crystal structures, and selecting pseudo sequence sites.

(1) Compute the distance between the MHC I molecule and the polypeptide: the general selection principle for the position distribution information of the binding groove is to select the positions corresponding to the amino acid residues in pMHCI[2], where the spatial distance between MHC I and the peptide is less than 4 Å by observing the structure of pMHCI. As shown in Methods 2.1, we compute the distance between the MHC I molecular residues and the peptide residues for each crystal structure, and record the mapping between the MHC I site and its corresponding peptide site in pMHC I that conform to the position sequence (Supplementary Table 1) relation.

Method 2.1 Extracts MHC I sites within 4.0Å of the peptide

Input: pMHCI structure <MHC I, peptide>
Output: A dictionary-style data structure *SiteDict* that records the mapping relationship between MHC I sites and peptide sites

Initialize MHC I molecular sequence length *L*
for *i* ← 1 to *L* **do**
for *j* ← 1 to 9 **do**
$d_{ij} = \sqrt{(X_{mhc_i} - X_{pep_j})^2 + (Y_{mhc_i} - Y_{pep_j})^2 + (Z_{mhc_i} - Z_{pep_j})^2}$
if d_{ij} < *4.0Å* **then**
SiteDict[j].append(i)
end if
end for
return *SiteDict*

(2) Align the MHC I site in the crystal structure: Since the MHC I sequence in the

pMHCI complex structure is missing and has different lengths, the sites will have difference between the MHC I sequence in the crystal structure and the preprocessed MHC I sequence. For this reason, this study employs the MAFFT[3] tool to carry out multiple sequence alignments for the MHC I sequence in the complex and the preprocessed MHC I sequence, and then we obtain the corrected complex structure of MHC I sequence. After that, we update the position of the MHC I site from SiteDict （Supplementary Table 1）.

(3) Selecting pseudo sequence site: To select the necessary sites from SiteDict, we count the distribution of the amino acid positions, and demonstrate the frequency distribution of occurrence sites (Supplementary Figure 1) and the distribution of the number of locations for pseudo sequences based on different occurrence frequency thresholds (Supplementary Figure 2). After that, we determine a frequency threshold as 57 according to the characteristics of the above-mentioned distribution graph. And then, only sites whose occurrence frequency value is greater than the cutoff value 57 can exist in the MHC I pseudo sequence sites. Due to the similarity of the amino acid sequences of the MHC I alleles, this study deletes the MHC-I sites with complete amino acid repeats by carrying out a consistent comparison of the candidate pseudo sequences. Finally, we have a 9*36 MHC-I pseudo sequence distribution matrix, listed by Supplementary Figure 3.

Supplementary Method 3 Degenerate coding

To generate a degenerate code, we first extract the pseudo sequence of the corresponding allele from the MHC-I pseudo sequence distribution according to the name of the input MHC I allele. The detailed process is shown in Supplementary Method 3.1. Then, we use Supplementary method 3.2 to generate one-hot encoding of the 9mer peptide obtained in Supplementary method 1. By combining one-hot encoding with pseudo sequences, we generate degenerate encodings (Supplementary Method 3.3).

Supplementary Method 3.1 Generate pseudo sequence

Method 3.1 Generate pseudo sequence

Input: MHC-I Allele names

Output: Corresponding pseudo sequence

mhc_sequence = [YY...WY] ← MHC-I sequence obtained by MHC-I Allele names
mhc_site = [7, 9 … 167, 171] ← MHC site obtained from the abscissa of Supplementary Figure3 MHC I pseudo sequence distribution
M = [[]] ← init pseudo sequence matrix
concat_site = {1:[7, 59, 62, 63, 159, 163, 167, 171], …, 9: [77, 80, 81, 84, 95, 97, 116, 123, 143, 146, 147]}← 9mer peptide and MHC contact sites obtained from the abscissa of Supplementary Figure3 MHC I pseudo sequence distribution
for *i* in range(1,9) **do**
    mhc_site_sequence = concat_site[i] ←The MHC site corresponding to peptide i
        for *i,aa* in enumerate(mhc_sequence) **do**
            append(*mhc_site_sequence, aa*) ←The corresponding amino acid in mhc_sequence will be extracted according to the position number of mhc_site
        mhc_site_sequence = set(mhc_site_sequence) ← Get amino acid types
        end for
        for *j* in enumerate(standard_amino_acid) **do** ← Traversing twenty standard amino acids, if there is such an amino acid, set it to 1
            if *j* in *mhc_site* **do**
                append(M[i], 1)
            else **do**
                append(M[i], 0)
            end if
        end for
end for
return *M* ← Returns a pseudo sequence matrix of size 9*20

Supplementary Method 3.2 One_hot encoding

Method 3.2 Encoding 9mer peptides using one hot

Input: 9mer peptide from 2.2.2

Output: One hot coding matrix

peptide ← 9mer peptide
M = [[]] ← One hot coding matrix of 9mer peptide
for *i,aa* in *enumerate(peptide)* **do**
        for *j* in enumerate(standard_amino_acid) **do** ← Traversing twenty standard amino acids
            if *aa* == *j* **do**
                append(*M[i]*, 0.9) ← Appended *v* to the collection of *V*

```
            else do
                append(M[i], 0.05)  ← Appended v to the collection of V
            end if
        end for
end for
return M  ← Returns the one hot encoding matrix of 9*20
```

Supplementary Method 3.3 Combining one-hot encoding with pseudo sequences

Input: One-hot encoding matrix and pseudo sequences matrix

Output: Degenerate coding

```
One_hot_Matrix  ← One-hot encoding matrix with shape of 9*20
Pes_seq_Matrix  ← pseudo sequences matrix with shape of 9*20
One_hot_Matrix = reshape(One_hot_Matrix ,(1,9,20))
Pes_seq_Matrix = reshape(Pes_seq_Matrix ,(20,9,1))
M = [[[]]]  ← Degenerate coding matrix M
for i,aa in enumerate(standard_amino_acid) do  ← Traversing twenty standard amino acids
        matrix_layer = concat(Pes_seq_Matrix[:,:,1] , One_hot_Matrix[i,:,:], axis = 3)  ←
Combining pseudo sequence and degenerate coding with axis = 3
        M = concat(M , matrix_layer, axis = 1)  ← concat with axis = 1
end for
return M  ← Returns the degenerate code matrix of 20*9*21
```

Supplementary Method 4 Weight initialization for Attention mechanism

Input: All MHC-I residue sequences

Output: Initialize the network

```
MHC_sequences  ← All MHC-I residue sequences
Amio_acid_frequency = {}  ← Amino acid frequency
Attention_Blocks  ← Attention_Blocks contains twenty depth convolution kernels
for seq in MHC_sequences do  ← Iterate over all MHC residues
    for amio_acid in seq do  ← Iterate all amio acid
        Amio_acid_frequency[amio_acid] +=1  ← Amio_acid_frequency indicates the number of such amino acids in MHC residues
    end for
end for
for key in Amio_acid_frequency.keys do  ← Traversing twenty amino acids
    init_weight = Eq. 3  ← Get the initialization value of neurons in each layer which detail show by Supplementary Metho 6
    Attention_Blocks[key] = Conv1d (init_weight)  ← All parameters of the attention_block layer corresponding to the amino acid key are set to init_weight by Eq. 4
return Attention_Blocks  ← Returns Attention_Blocks
```

Supplementary Method 5 Training data augmentation by Semi-supervised learning

Input: Mass spectrometry data: Peptide and allel

Output: Pre-trained ConvNeXt_BA model for semi-supervised learning using NetMHCpan 4.1 to predict correct data and its affinity values

pep ← Peptide Sequence
hla = [] ← HLA Sequence
ms = [] ← Mass spectrometry answer
Predict_ V = [] ← Save the data where predictions match experimental results
for *i,j,a* in *pep,hla,ms* **do**
    l*og50k* = NetMHCpan4.1(*i, j, a*) ← Generate NetMHCpan4.1 BA prediction's value
    *Predict_ic50 =10^((1- log50k)*log(50000))* ← Convert log50k to ic50
    label = Eq.6(*Predict_ic50*) ← Generate NetMHCpan4.1 BA prediction's value
    if *label == a* **do**
        append(*Predict_ V*, *(i, j, Predict_BA)*) ← Appended *v* to the collection of *V*
    end if
end for
Pretrain_BAmodel = ConvNeXt_BA() ← Inti ConNext-MHC_BA model
for *pep, hla, Predict_BA* in *Predict_ V* **do**
    Matrix = degenerate_code_matrix(*pep,hla*)
    Pretrain_BAmodel.fit(*Matrix* , *Predict_BA*)

Supplementary Method 6 Prior knowledge of the normalized frequency values of twenty amino acids in residues.

Input: 159 MHC I residues

Output: Normalized frequency values of twenty amino acids

Initialize amino frequence *frequence_amino* = {'P': 0,'...,Y':0}
Initialize amino weight *amino weight* = {'P': 0,'...,Y':0}
Initialize residues *MHC_residues* , residues length *L*,
for *i* ← 1 to 159 **do**
    for *j* ← 1 to *L* **do**
        frequence_amino[*MHC_residues*[*i*][*j*]]+=1
    end for
end for
for *i* ← 20 aminos **do**

$$amino\_weight[i] = \frac{frequence\_amino[i] - Min(frequence\_amino)}{Max(frequence\_amino) - Min(frequence\_amino)}$$

end for
return *amino_weight*

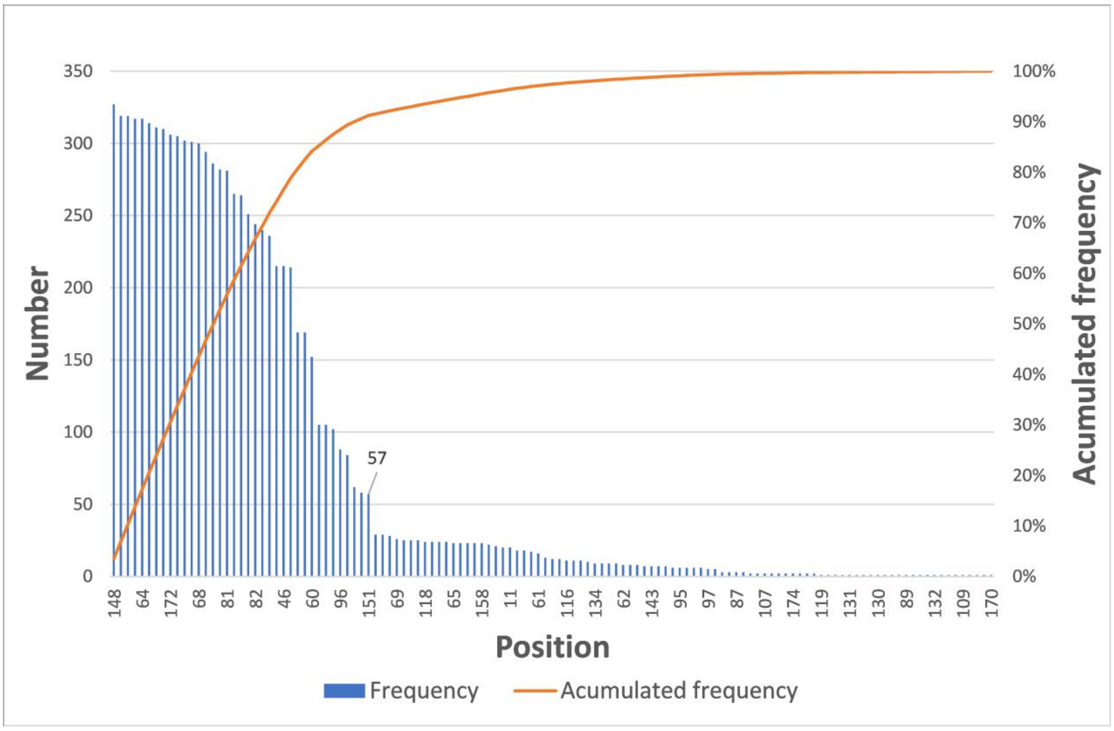

Supplementary Figure 1 Occurrence frequency in pseudo sequence positions

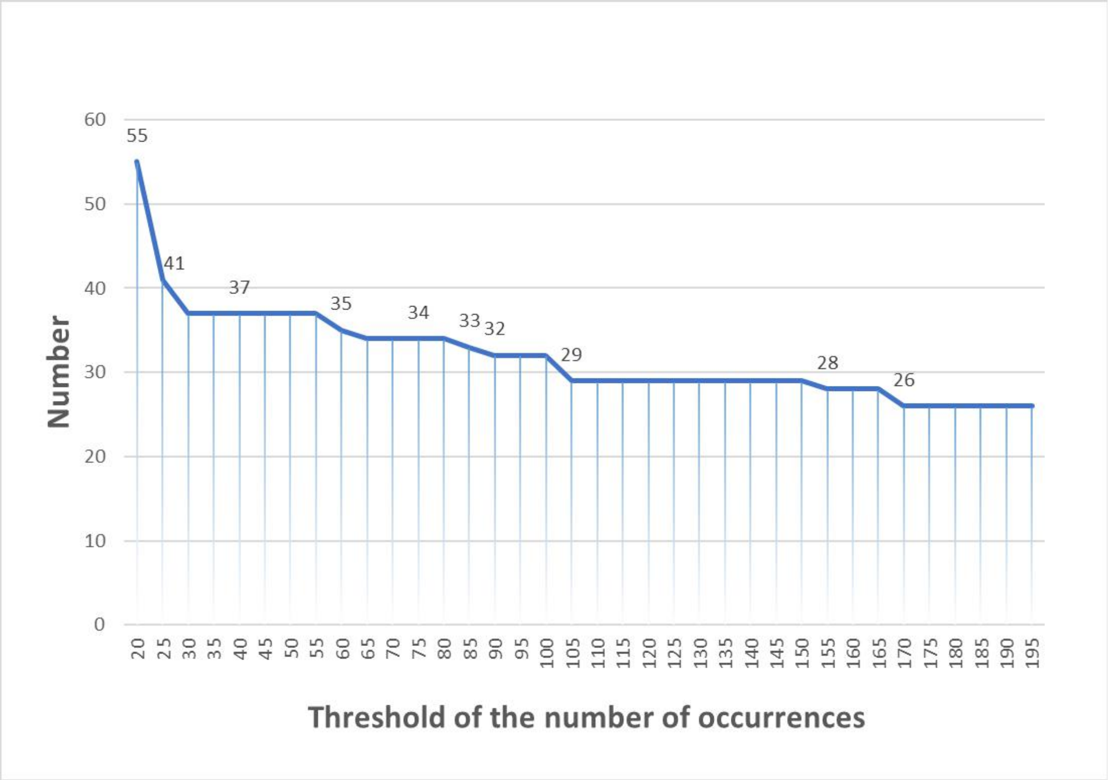

Supplementary Figure 2 Pseudo sequence position number distribution

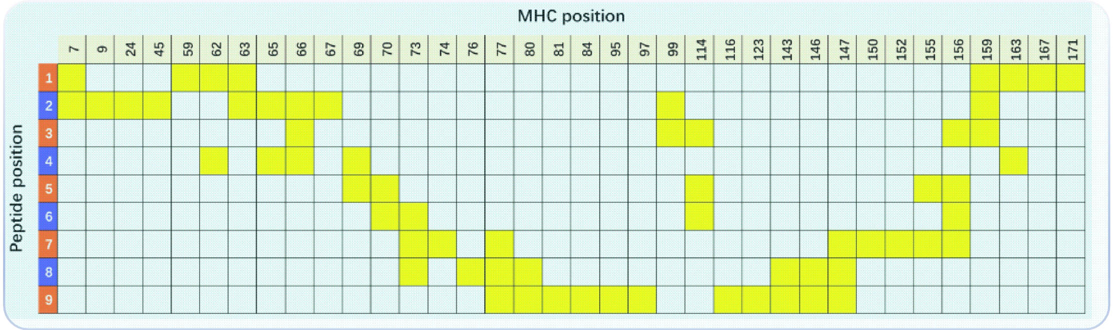

Supplementary Figure 3 MHC I pseudo sequence distribution

The abscissa represents the 9mer peptide site, the ordinate represents the MHC I site, and the yellow grid represents whether the MHC I molecule is in contact with the peptide. For example, the first line represents that the first amino acid of the 9mer peptide is in contact with residues 7, 59, 62, 63, 159, 163, 167, and 171, which is represented as 1.

Therefore, by inputting a specific MHC sequence, we can obtain the specific residues corresponding to each site of the 9mer peptide structure, and then annotate the types of amino acids which the site contacts with. We use a 9*20 matrix to represent the Pesudo-sequence by Figure 2a, where 9 represents the length of the peptide, 20 represents the type of residue amino acid, 1 represents that this position is in contact with the corresponding amino acid, and 0 represents no contact.

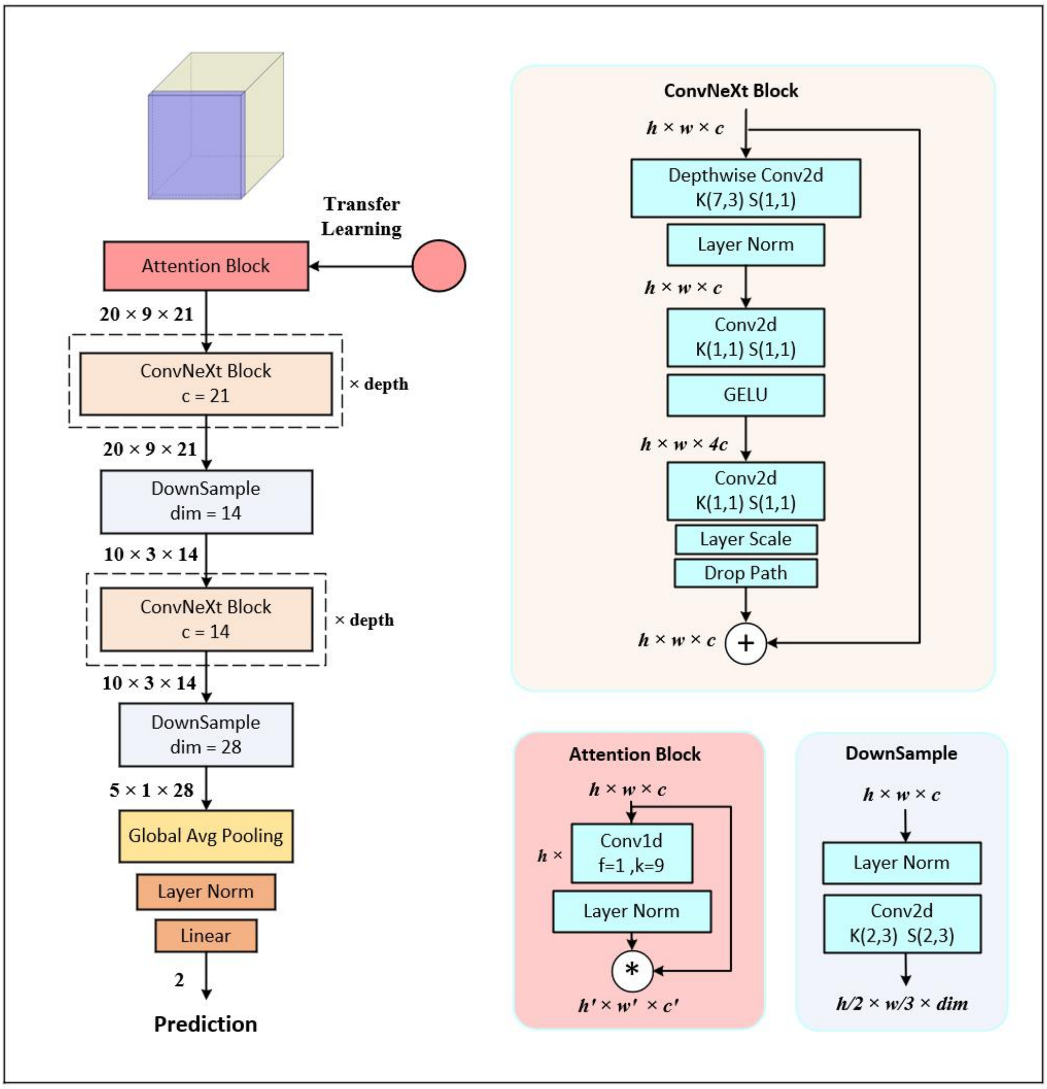

Supplementary Figure 4 ConvNeXt-MHC model structure design

Supplementary Figure 4 shows that our ConvNeXt-MHC model consists of an initial Attention Block, two layers of ConvNeXt Block [4], and DownSample layer.

First, the Attention Block computes 20 weight coefficients (attention) of the input matrix (20*9*21) in its first dimension, and implements the attention mechanism by multiplying with the matrix.

Subsequently, we employ the first ConvNeXt Block to do feature extraction for the input matrix (20*9*21). Since ConvNeXt_Block uses a residual network design, the data size of the module will be maintained. Next, downsampling is carried out by the DownSample layer for feature fusion. Subsequently, the same feature extraction and fusion steps are performed, and the MLP layer is finally used to output the prediction score.

The attention mechanism is used to further obtain the influence of the amino acid species of the residues on the 9mer peptide. As shown in Figure 3a, neurons are added to each layer of matrix to obtain the specific weight of the layer. Since in the matrix design, the degenerate coding only represents the contact information between 9mer peptide and specific amino acid species, and the number of specific amino acids is lost, so we use transfer learning based on prior knowledge to count all MHC-I residues in Eq. 2. The proportion of each type of amino acid is normalized as the initialization weight of the network (Supplementary Method 6).

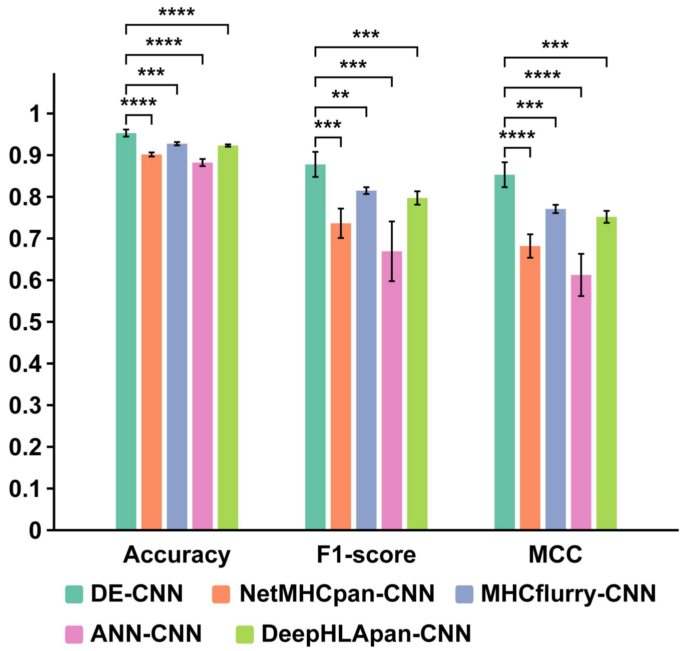

Supplementary Figure 5 Comparison among encoding methods of DE-CNN, NetMHCPan4.1, MHCflurry2.0.1, ANN4.0 and DeepHLApan

After we use independent samples T-test to compute the p-value, we use an asterisk (*) to indicate the significance level. The specific rules are as follows:

An asterisk (*): Usually indicates a p-value less than 0.05, indicating that the difference is statistically significant.

Two asterisks (): ** usually indicate that the p-value is less than 0.01, indicating that the difference is highly statistically significant.

Three asterisks (*): *** usually indicates that the p-value is less than 0.001, indicating that the difference is statistically significant.

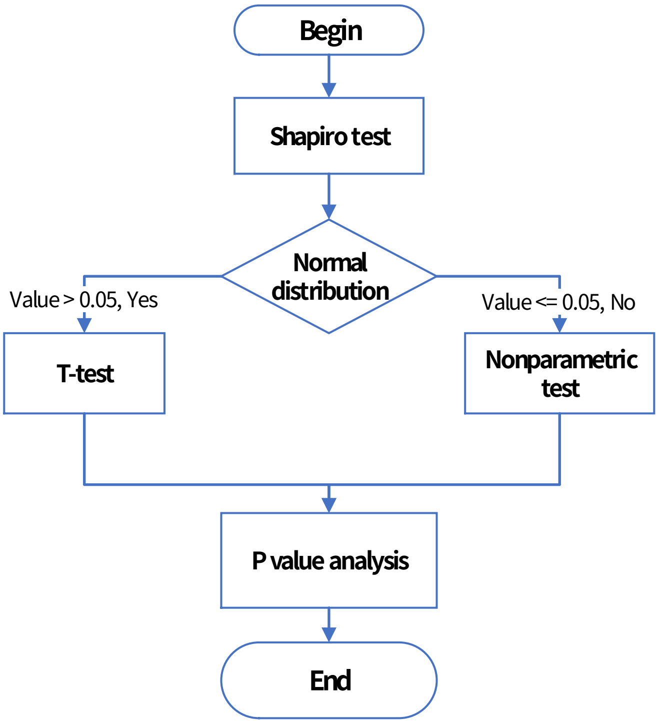

Supplementary Figure 6 Work flow of the statistical test

Then we carry out shapiro test and t-test for the accuracy of Supplementary Table 3. The shapiro test will confirm if the original data conform to a normal distribution. As shown in Table 1, the shapiro test p-values of DE-CNN, NetMHCpan-CNN, MHCflurry-CNN, DeepHLApan-CNN and ANN-CNN are 0.710, 0.586, 0.050, 0.457 and 0.431, respectively, which are greater than the cutoff value of 0.05, indicating that the five types of results conform to the normal distribution.

Under the normal distribution condition, we carry out T-test for these MHC-I predictive applications, DE-CNN, NetMHCpan-CNN, MHCflurry-CNN, DeepHLApan-CNN and ANN-CNN. The assumption: H_0: $\mu_1 = \mu_2$, the alternative hypothesis is: H_1: $\mu_1 \neq \mu_2$, where μ_1 and μ_2 are the averages of the two types of samples. As shown in Table 1, since both T-tests are less than the cutoff value of 0.05, it indicates the statistically difference between DE-CNN and other models.

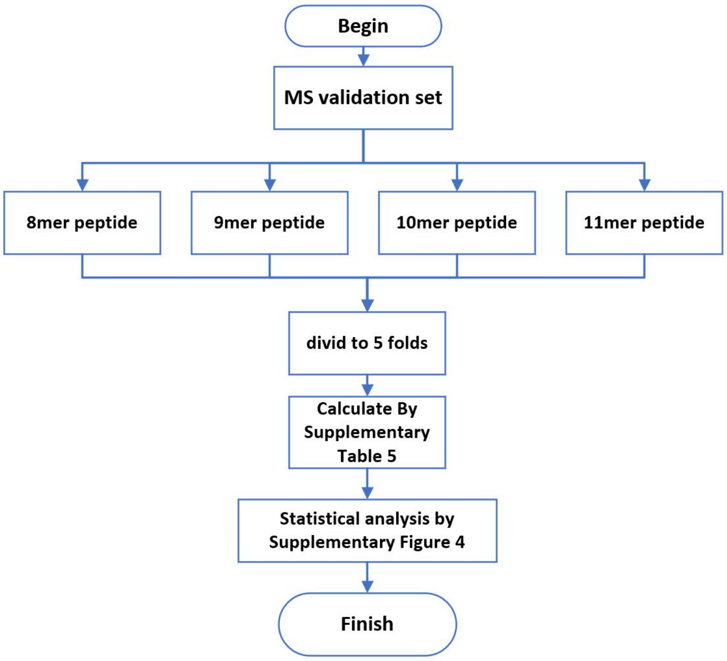

Supplementary Figure 7 T-test process of different models on MS validation set

To effectively compute the differences between different models, we divided the MS validation set into four categories based on peptide length: 8, 9, 10, and 11. At the same time, each type of data is randomly divided into five folds, and the accuracy, f1-score, and MCC of each fold are computed according to the formula of Supplementary Table 5. Statistical analysis of the five fold's results is carried out according to the process of Supplementary Figure 6, and the results are summarized in Supplementary Table 6.

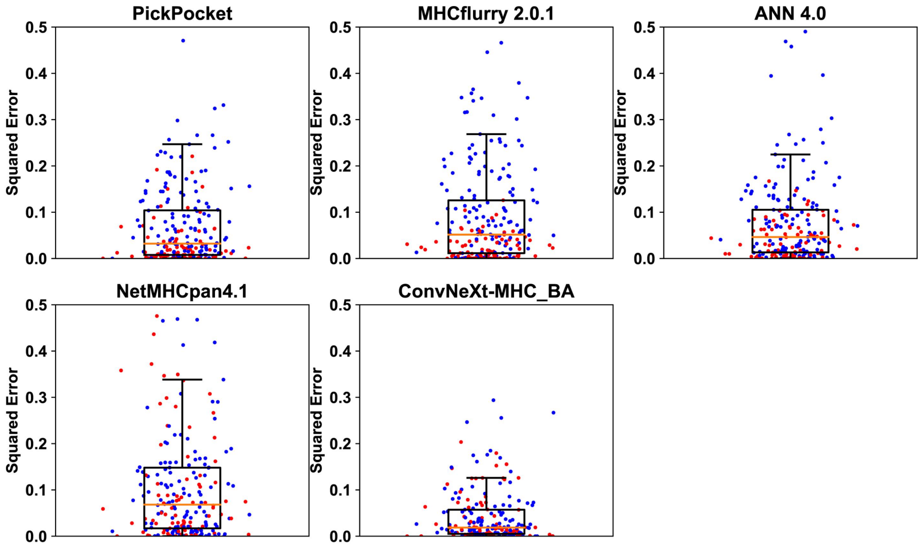

Supplementary Figure 8 The performance comparison among ConvNeXt-MHC_BA, PickPocket, ANN4.0, NetMHCpan4.1 and MHCflurry2.0.1 using AF validation set.

Here, the data of the Supplementary Figure 8 was collected from the recently

released weekly IEDB data (Supplementary Figure 9).

Supplementary Figure 8 plots the prediction results for each model. The red dots and blue dots represent binding and non-binding respectively.

Supplementary Figure 8 demonstrates that ConveNeXt-MHC_BA's squared errors (Supplementary Table 4) are more concentrated than other models, and its mean value is more statistically significant than other models (Supplementary Table 6), indicating ConvNeXt-MHC_BA outperforms other models.

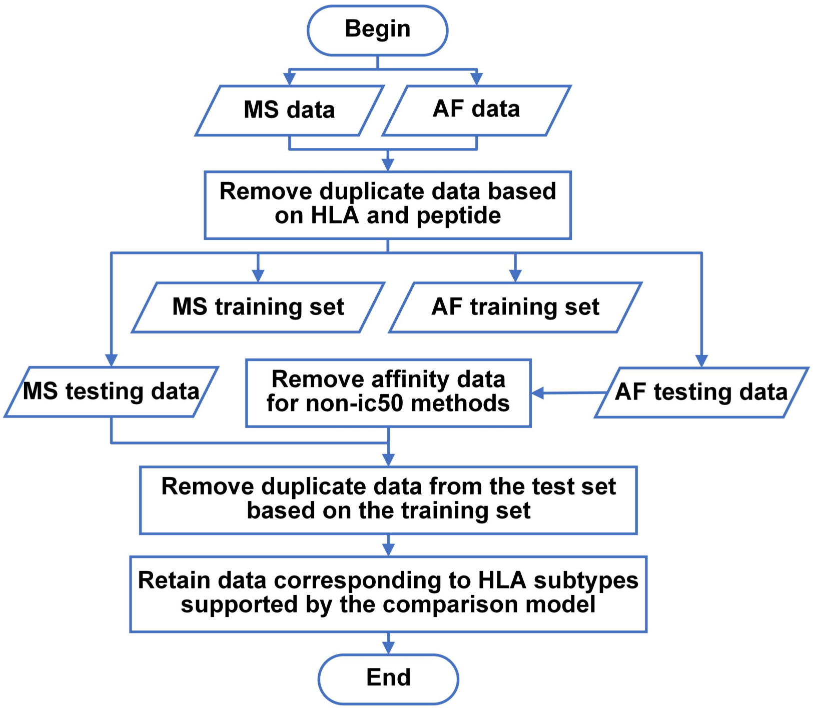

Supplementary Figure 9 Processing data set flow chart.

Supplementary Table 1. Nomenclature

mhc_i	The first i site of MHC-I
pep_j	The jth position of the polypeptide
d_{ij}	The direction between mhc_i and pep_j
X_{mhc_i}, Y_{mhc_i}, Z_{mhc_i}	X, Y, Z coordinates of mhc_i
X_{pep_j}, Y_{pep_j}, Z_{pep_j}	X, Y, Z coordinates of pep_j
Position Sequence	Structurally observe and select sites in pMHCI corresponding to amino acid residues whose spatial distance

	between MHC I and polypeptide is less than 4Å
SiteDict	The mapping relationship between the MHC I site corresponding to the position sequence and its corresponding polypeptide site in pMHC-I
ILA	we designed two-dimensional image-like arrays (ILAs) to represent MHC-I pseudo sequences and 9-mer peptide sequences
$DepthConv(V_c, X_{[:,:,c]})$	Depthwise Convolution: This is a type of convolutional layer commonly used in conjunction with pointwise convolution layers to form a Depthwise separable convolution. It's an efficient way to process data with high channel dimension
	Among them, $X = [X_{[:,:,1]}; \ldots; X_{[:,:,C]}]$, matrix *X* represents the input matrix; C represents the number of channels in the third dimension of the input matrix *X*, c∈C, V_c represents the convolution kernel corresponding to the c channel, K and L represent the convolution kernel sizes K and L respectively, k∈K, l∈L, *i* and *j* represent the positions of the first two dimensions in the input matrix *X* Value; DepthConv represents depth convolution calculation, WiseConv represents point-by-point convolution calculation.
init_weight[h](Eq.3)	Among them, $frequency_{Amio\_acid}[h]$ represents the frequency of twenty amino acids, h represents one of the twenty amino acids, and *init_weight* represents the initial weight of the attention block;
TN	The number of true negative
TP	The number of true positive
FN	The number of false negative
FP	The number of false positive
P	The number of positive

N	The number of negative

Supplementary Table 2. Pseudo sequence position sequence

Model	Pseudo Positon List
NetMHCpan4.1	7, 9, 24, 45, 59, 62, 63, 66, 67, 69, 70, 73, 74, 76, 77, 80, 81, 84, 95, 97, 99,114, 116, 118, 143, 147, 150, 152, 156, 158, 159, 163, 167, 171
MHCflurry2.0.1	7, 9, 24, 45, 59, 62, 63, 66, 67, 69, 70, 73, 74, 76, 77, 80, 81, 84, 91, 95, 97,99, 102, 114, 116, 118, 143, 147, 150, 152, 156, 158, 159, 163, 167, 171, 199
ConvNeXt-MHC	7, 9, 24, 45, 59, 62, 63, 65, 66, 67, 69, 70, 73, 74, 76, 77, 80, 81, 84, 95, 97,99, 114, 116, 123, 143, 146, 147, 150, 152, 155, 156, 159, 163, 167, 171

Supplementary Table 3. Comparison of five-fold cross test performance of models based on different pseudo sequence encodings

Fold	Method	Accuracy	Precision	Recall	F1-score	MCC
0	MHCflurry-CNN	0.92885	0.81701	0.82414	0.82056	0.77620
	NetMHCpan-CNN	0.89472	0.85047	0.56620	0.67981	0.63820
	DE-CNN	0.94172	0.96516	0.73114	0.83201	0.80882
	DeepHLApan-CNN	0.92557	0.83607	0.77334	0.80349	0.75854
	ANN-CNN	0.88381	0.78740	0.56094	0.65515	0.59986
1	MHCflurry-CNN	0.92067	0.78333	0.82480	0.80353	0.75426
	NetMHCpan-CNN	0.90676	0.77362	0.74357	0.75830	0.70078
	DE-CNN	0.95167	0.93926	0.80642	0.86778	0.84207
	DeepHLApan-CNN	0.92264	0.78359	0.83851	0.81012	0.76231
	ANN-CNN	0.86897	0.84896	0.40654	0.54980	0.52910
2	MHCflurry-CNN	0.93037	0.87147	0.75854	0.81109	0.77145
	NetMHCpan-CNN	0.90089	0.80235	0.65954	0.72397	0.66902
	DE-CNN	0.96246	0.88765	0.92680	0.90680	0.88363
	DeepHLApan-CNN	0.92247	0.83871	0.74822	0.79089	0.74531
	ANN-CNN	0.88991	0.72608	0.70370	0.71471	0.64665
3	MHCflurry-CNN	0.92775	0.79345	0.85824	0.82457	0.78012
	NetMHCpan-CNN	0.90592	0.76944	0.74896	0.75906	0.70072
	DE-CNN	0.96036	0.88586	0.91795	0.90161	0.87702
	DeepHLApan-CNN	0.92589	0.81836	0.80111	0.80964	0.76370
	ANN-CNN	0.87978	0.66372	0.78841	0.72071	0.64872
4	MHCflurry-CNN	0.93005	0.84775	0.78420	0.81474	0.77260
	NetMHCpan-CNN	0.90008	0.71571	0.81376	0.76159	0.70102
	DE-CNN	0.94899	0.81220	0.96245	0.88096	0.85375
	DeepHLApan-CNN	0.91911	0.86284	0.69892	0.77228	0.72969
	ANN-CNN	0.88884	0.73374	0.68048	0.70610	0.63837

Supplementary Table 4. Five classic classification measurement standards, detailed by supplementary Table 1

Measure	Formula
$Accuracy$	$\frac{TP+TN}{P+N}$
$Precision$	$\frac{TP}{TP+FP}$
$Recall$	$\frac{TP}{TP+FN}$
F1-score	$2*\frac{Precision*Recall}{Precision+Recall}$
MCC	$\frac{TP\times FP-FP\times FN}{\sqrt{(TP+FP)(TP+FN)(TN+FP)(TN+}}$
$log50k$	$1-\frac{log\,(ic50)}{log\,(50000)}$
$Squared\ Error$	$(Y_{ture}-Y_{predicaion})^2$

Supplementary Table 5. T-test answer of different models on MS validation set

Peptide length	Methods	Accuracy	F1-score	MCC	Accuracy's STD	F1-score's STD	MCC's STD	Shaprio p-value	T-test p-value
8mer peptide	BigMHC	0.5730	0.3234	0.3156	0.0671	0.1393	0.0931	0.6557	0.0005
	PickPocket	0.4757	0.0367	0.0585	0.0582	0.0452	0.0723	0.0707	0.0001
	ANN4.0	0.4973	0.1221	0.1282	0.0471	0.0795	0.0792	0.7583	0.0000
	NetMHCpan4.1	0.5459	0.2535	0.2178	0.0551	0.1615	0.1503	0.2279	0.0003
	MHCflurry2.0.1	0.4973	0.1130	0.1001	0.0367	0.1136	0.1009	0.4925	0.0000
	DeepHLApan	0.5784	0.7154	0.2273	0.0737	0.0584	0.0795	0.1896	0.0015
	DE-CNN	0.6432	0.4990	0.4029	0.0791	0.1442	0.1538	0.2032	0.0123

	ConvNext-MHC_BA	0.8054	0.7746	0.6634	0.0626	0.0801	0.0998	0.3758	1.0000
9mer peptide	BigMHC	0.7300	0.6358	0.5443	0.0070	0.0295	0.0180	0.2213	0.0000
	PickPocket	0.6603	0.5023	0.4275	0.0151	0.0308	0.0231	0.5833	0.0000
	ANN4.0	0.7866	0.7385	0.6237	0.0146	0.0187	0.0200	0.4999	0.0000
	NetMHCpan4.1	0.8795	0.8688	0.7730	0.0057	0.0057	0.0073	0.7213	0.0014
	MHCflurry2.0.1	0.8531	0.8351	0.7279	0.0100	0.0122	0.0156	0.1510	0.0001
	DeepHLApan	0.8735	0.8672	0.7522	0.0097	0.0105	0.0181	0.2568	0.0014
	DE-CNN	0.8876	0.8795	0.7855	0.0091	0.0087	0.0155	0.9156	0.0274
	ConvNext-MHC_BA	0.9048	0.9005	0.8143	0.0089	0.0098	0.0158	0.2153	1.0000
10mer peptide	BigMHC	0.7639	0.6188	0.5618	0.0279	0.0324	0.0330	0.6952	0.0002
	PickPocket	0.6238	0.2100	0.2503	0.0203	0.0555	0.0485	0.8202	0.0000
	ANN4.0	0.8138	0.7283	0.6272	0.0277	0.0766	0.0758	0.9595	0.0048
	NetMHCpan4.1	0.8522	0.7961	0.7065	0.0152	0.0332	0.0342	0.2403	0.0463
	MHCflurry2.0.1	0.8234	0.7413	0.6576	0.0159	0.0417	0.0396	0.4490	0.0033
	DeepHLApan	0.7677	0.7664	0.5716	0.0446	0.0539	0.0741	0.1133	0.0017
	DE-CNN	0.8935	0.8712	0.7801	0.0256	0.0370	0.0558	0.2764	0.8443
	ConvNext-MHC_BA	0.8896	0.8681	0.7732	0.0279	0.0406	0.0605	0.0775	1.0000
11mer peptide	BigMHC	0.6900	0.5573	0.4779	0.0116	0.0682	0.0350	0.7540	0.0000
	PickPocket	0.5450	0.2015	0.2333	0.0406	0.0131	0.0231	0.5033	0.0000
	ANN4.0	0.6700	0.5281	0.4529	0.0357	0.0339	0.0290	0.1117	0.0000
	NetMHCpan4.1	0.8325	0.8092	0.6951	0.0275	0.0313	0.0425	0.2707	0.0270
	MHCflurry2.0.1	0.7875	0.7417	0.6304	0.0328	0.0345	0.0440	0.6991	0.0016
	DeepHLApan	0.5600	0.6942	0.1970	0.0376	0.0367	0.0638	0.6627	0.0000
	DE-CNN	0.8638	0.8613	0.7277	0.0254	0.0276	0.0494	0.0857	0.3407
	ConvNext-MHC_BA	0.8813	0.8825	0.7606	0.0234	0.0260	0.0460	0.2574	1.0000

Supplementary Table 6. Wilcoxon test answer of different models on AF's MSE value of validation set

Model name	Mean	Std	Shapiro	Wilcoxon(P-value)
PickPocket1.1	0.067	0.080	7.19E-16	0.00006
MHCflurry2.0.1	0.087	0.099	3.47E-15	0.00000
ANN4.0	0.075	0.087	6.91E-17	0.00000
NetMHCpan4.1	0.107	0.125	2.83E-16	0.00000
ConvNext-MHC_AP	0.041	0.055	2.49E-18	1.00000

Supplementary Table 7. Five-fold cross-validation results of ConvNeXt-MHC_AP on MS training data set

Folds	Accuracy	Precision	F1-score	Recall	MCC
1	0.964	0.907	0.908	0.910	0.886
2	0.964	0.906	0.908	0.909	0.885
3	0.963	0.908	0.907	0.905	0.884
4	0.964	0.910	0.909	0.908	0.887
5	0.965	0.908	0.911	0.914	0.889
Means	0.964	0.908	0.909	0.910	0.886

Supplementary Table 8. Five-fold cross-validation results of ConvNeXt-MHC_AP and DE-CNN on MS data set

5folds	Method	Accuracy	F1-score	MCC
Mean	DE-CNN	0.953	0.877	0.853
	ConvNeXt-MHC_AP	0.964	0.909	0.886
	Promotion rate	1.10%	1.04%	1.04%

Reference

1. Reynisson B, Alvarez B, Paul S et al. NetMHCpan-4.1 and NetMHCIIpan-4.0: improved predictions of MHC antigen presentation by concurrent motif deconvolution and integration of MS MHC eluted ligand data, Nucleic Acids Res 2020;48:W449-W454.
2. Berman HM, Westbrook J, Feng Z et al. The Protein Data Bank, Nucleic Acids Res 2000;28:235-242.
3. Katoh K, Standley DM. MAFFT multiple sequence alignment software version 7: improvements in performance and usability, Mol Biol Evol 2013;30:772-780.
4. Liu Z, Mao H, Wu C-Y et al. A convnet for the 2020s. In: Proceedings of the IEEE/CVF conference on computer vision and pattern recognition. 2022, p. 11976-11986.
